# New pre-arrival instructions can avoid abdominal hand placement for chest compressions

**DOI:** 10.1186/1757-7241-21-47

**Published:** 2013-06-22

**Authors:** Tonje S Birkenes, Helge Myklebust, Jo Kramer-Johansen

**Affiliations:** 1 Institute for Experimental Medical Research, Oslo University Hospital and University of Oslo, Ulleval, PO Box 4956 Nydalen, N-0426 OSLO, Norway; 2 Laerdal Medical AS, Tanke Svilandsgate 30, N-4002 Stavanger, Norway

## Abstract

**Objective:**

To investigate if modified pre-arrival instructions using patient’s arm and nipple line as landmarks could avoid abdominal hand placements for chest compressions.

**Method:**

Volunteers were randomized to one of two telephone instructions: “Kneel down beside the chest. Place one hand in the centre of the victim’s chest and the other on top” (control) or “Lay the patient’s arm which is closest to you, straight out from the body. Kneel down by the patient and place one knee on each side of the arm. Find the midpoint between the nipples and place your hands on top of each other” (intervention). Hand placement was conducted on an adult male and documented by laser measurements. Hand placement, quantified as the centre of the compressing hands in the mid-sagittal plane, was compared to the inter-nipple line (INL) for reference and classified as above or below. Fisher’s exact test was used for comparison of proportions.

**Results:**

Thirty-six lay people, age range 16–60, were included. None in the intervention group placed their hands in the abdominal region, compared to 5/18 in the control group (p = 0.045). Using INL as a reference, the new instructions resulted in less caudal hand placement, and the difference in mean hand position was 47 mm [95% CI 21,73], p = 0.001.

**Conclusion:**

New pre-arrival instructions where the patient’s arm and nipple line were used as landmarks resulted in less caudal hand placements and none in the abdominal region.

## Background

Initiation of bystander CPR doubles the chance of survival after out-of-hospital cardiac arrest and is probably the most feasible intervention to improve overall survival in many communities [1]. CPR pre-arrival instructions resulted in 50% increase in bystander CPR in King County [2,3], and is recommended by American Heart Association [4].

In the first publication on chest compressions for cardiac arrest in humans, Kouwenhoven described hand placement as “the heel of one hand […] is placed on the sternum just cephalad to the xiphoid” [5]. Subsequent CPR guidelines have retained the lower half of the sternum as the target, with different combinations of anatomical landmarks in the instructions to achieve this.

“Place the heel of the hand on the lower half of sternum” was used from 1966 to 1974 [6,7]. In 1980 the instructions changed to: “.. use the lower margin of the victim’s rib cage to find the notch where the ribs meet the sternum” [8]. Guidelines 2000 continued to use the notch, but introduced “the center of the chest between the nipples” as alternative simplified instructions [9]. In 2005 the instruction were simplified to “in the middle of the chest”, but the American guidelines recommended “in the middle of the chest, between the nipples” [10]. Both American and European guidelines have used “in the middle of the chest” since 2010 [11,12].

These instructions have resulted in a significant rate of incorrect or too low hand placement on the sternum and even in the abdominal region during lay people manikin CPR [13]–[18]. When we tested adult lay people CPR on a dressed manikin, almost half of the participants initially compressed in the abdominal region [17].

In this study, we wanted to test a new combination of anatomical landmarks aimed at avoiding abdominal hand placements. To make it more realistic, we used a dressed, adult person playing the role of the patient.

## Method

### Study design

During pilot testing, we identified sitting astride the arm and using internipple line (INL) as the best combination of landmarks to avoid abdominal hand placement.

We then conducted a randomized study to compare our new instruction set with ERC’s recommendation. Following consent, participants drew from a bowl with closed envelopes (50/50 control and intervention), without replacement. Randomization result was communicated to dispatcher by researcher on telephone. The study is registered locally at Oslo University Hospital with project number 2010/1519.

### Participants

We recruited volunteers among employees at the local Norwegian Labor and Welfare office (NAV) and from a youth group from The Norwegian Trekking Association, excluding participants with current or recent duty-to-respond. Written consent was obtained from all participants. Volunteers from NAV were offered a free CPR course in return for their participation.

### Test situation

The study was conducted in a well-lit room with two researchers present. The patient was played by a 46 year old 1.87 m tall adult male weighting 93 kg with sternal length 22.5 cm (from xiphosternal junction to jugular notch lying on the floor, wearing three layers of clothes (underwear, shirt, and sweater). The scenario was explained to participant: “*This person has a confirmed cardiac arrest and needs chest compressions*. *You must follow the instructions given by the dispatcher on the phone*. *To save time*, *you decide not to remove any clothes*”. All participants were handed a phone with an established connection with the dispatcher and speaker function activated, with the initial instruction: “*Place the phone on the floor in front of you such that we can hear each other*”. The person acting as dispatcher was located in another facility and provided instructions according to randomization, with telephone as the only source of communication. Two-way communication was possible, and a few participants asked the dispatcher to repeat the instructions once.

Each instruction set had two parts; to position the rescuer next to the victim and placement of the hands on the chest.

Instructions for the control group (based on ERC recommendations):

“Kneel beside the chest. Place the heel of your hand in the center of the chest with the other on top”

Instructions for the intervention group:

“Lay the patient’s arm which is closest to you, straight out from the body. Kneel down by the patient and place one knee on each side of the arm. Find the midpoint between the nipples and place your hands on top of each other.”

The test was ended when the participant had placed the hands on the chest. Since we used an adult playing the role of the patient and not a manikin, the participants were not asked to perform chest compressions.

### Hand placement measurement

**Figure 1:**
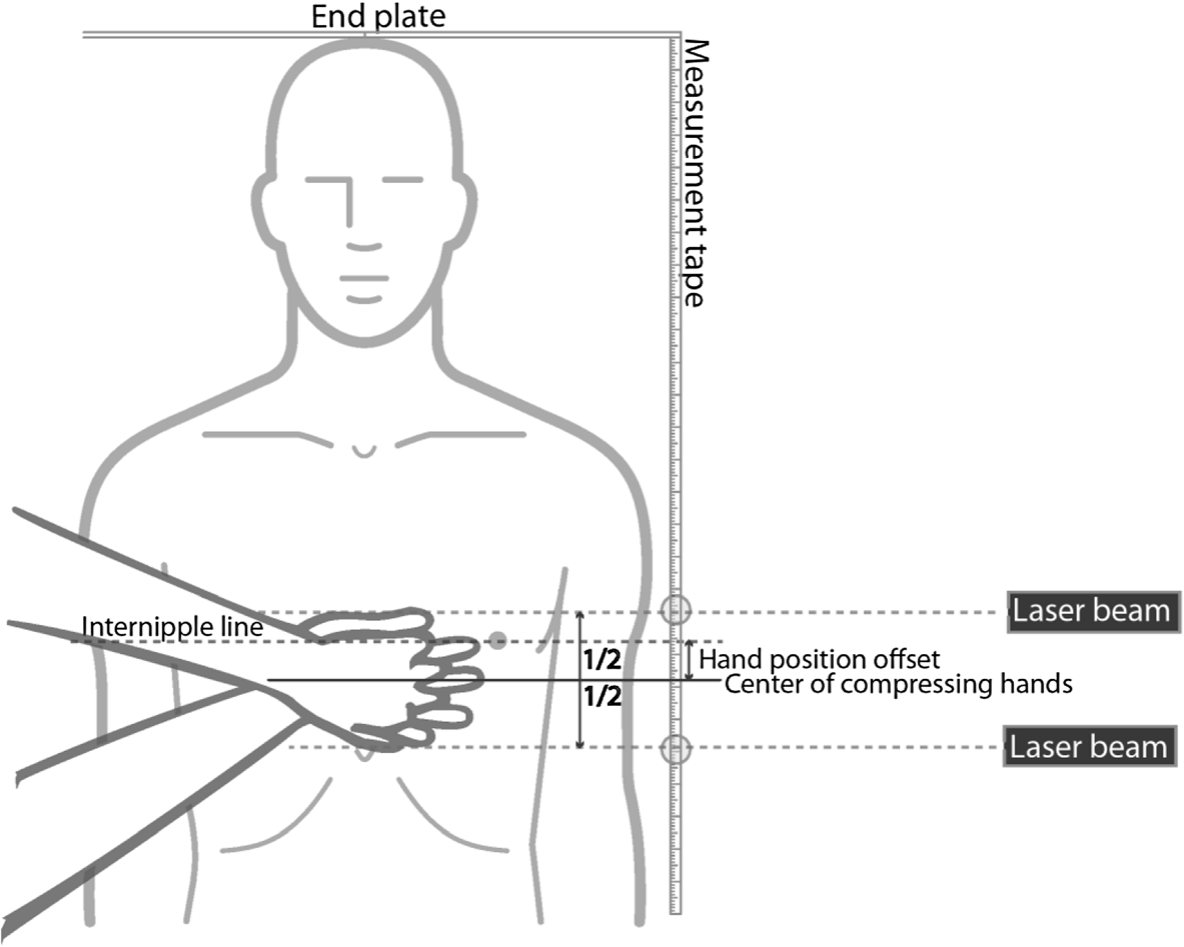
**Test arrangement and measurement principle.** Circle (○) indicates laser-ruler intersection for upper and lower border of compressing hands documented by photo.

Hand placement was measured by using a hard base with an end plate, measurement tape, laser beam (Black & Decker LZR6) and a digital camera (Nikon D40x). The marker was lying with the head against the end plate and the laser beam direction adjusted to be parallel to his INL. The hand position was measured using the laser beam at the upper and lower borders of the compressing hands and photographed. Hand position offset was quantified as the distance from the end plate to the center of the compressing hands in the mid-sagittal plane, compared to INL and classified as above or below. Negative offset value indicates hand placement caudal to INL. Hand position caudal to the xiphosternal junction was classified as abdominal. The laser beam was turned off until the participants had placed their hands. The researcher was blinded for the randomization when measuring the hand position from the photographs. See Figure 1 for test arrangement and measurement principle.

### Statistical analysis

Fisher’s exact test was used for comparison of proportions above and below INL. Un-paired t-test was used for continuous measurements (hand placement offset).

### Power analysis

The study was powered based on categorical pilot data where we found that only 14% placed their hands on the internipple line (or cephalad to INL). A power analysis to detect a change from 14% to 60%, a power (1- β) of 0.8 and a significance level (α) of 0.05, estimated to 17 test persons in each group.

## Results

Thirty-eight lay people between 16–60 years old were invited and signed up for the study, which was conducted in Stavanger (Norway), April 2010.

One control group subject placed the “patient’s” hands in the center of his chest, instead of her own. Hence no hand placement data were recorded due to this misunderstanding of instructions. One other subject declined participation on the study day, without giving specific reason.

No participant had any recent obligations as first-responders or professional responders to cardiac arrest. Demographic data of the remaining 37 participants is described in Table 1.

**Table 1:** **Demography**, **education and CPR training background of participants**

**Participants**	**Control group**	**Intervention group**
	**n = 19**	**n = 18**
Women	16	13
Age <20 years	5	5
Age ≥20 years	14	13
Completed education		
High school	5	5
Occupational school/lower university grade	7	6
Higher university grade	6	7
Previous CPR training		
Never attended CPR training	3	6
Completed 1–2 CPR courses	14	6
Completed more than 2 courses	2	5
Unknown	0	1
Years since last training:		
1-3 years	6	5
4-6 years	3	2
7-9 years	2	2
9+ years	5	2
Unknown	3	7

**Figure 2:**
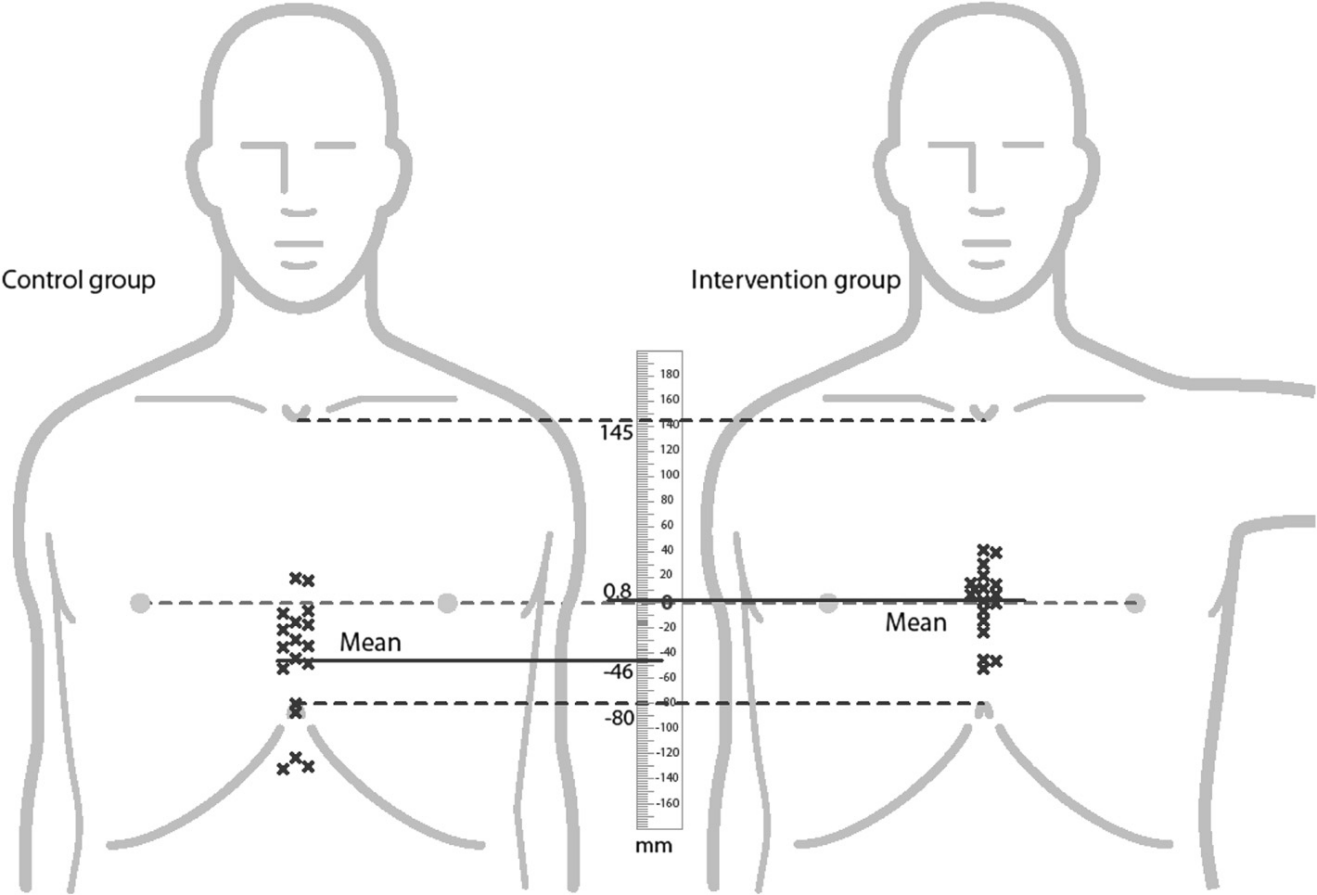
**Hand placement in the control and intervention groups.** Individual compression point centers are marked by x, the means by a full line, top and bottom of the sternum and inter-nipple line by interrupted lines.

Using the arm and internipple line as reference, the new instruction set resulted in less caudal hand placement, and the difference in mean hand position offset was 47 mm [95% CI 21,73], p = 0.001. None in the intervention group placed their hands in the abdominal region vs. 5/18 in the control group (p = 0.045) (Table 2 and Figure 2).

**Table 2:** **Hand placement relative to internipple line** (**INL**) **or over abdomen**

**Hand placement**	**Reference instructions**	**Intervention instructions**	
	**[n = 18]**	**[n = 18]**	
On/above INL	2	11	
Below INL	11	7	
Abdominal	5	0	
**Hand placement Relative to INL**			**Difference**
Mean offset* [mm]	−46 (±47)	0.8 (±28)	47 [21, 73], p = 0.001

* Minus sign indicates offset value caudal to INL. See “Hand placement measurement” in Methods section for definition.

Results for hand placement. Mean offset is the distance between center of compressing hands and internipple line.

## Discussion

The instructions of kneeling astride the arm and placing the hands between the nipples gave less abdominal hand placement compared to the instruction of placing them in the center of the chest. One of the reasons is probably that when sitting astride the arm the distance to the chest is short with a longer distance to the abdomen. Hand placement also varied less when using clear anatomical landmarks (arm and nipple line) compared to the center of the chest, which we think is more open for interpretation.

Improving hand placement for chest compressions is important since all the various instructions for hand placement used in the last decade [12,19,20], have resulted in poor hand placement [14,15,18,21]–[23], with a significant proportion of abdominal hand placement [16]–[18].

### Kneeling astride the arm, hand placement between the nipples, or both?

Both instruction sets comprises two instructions, and both instructions in the intervention instruction set are new. Introducing two new instructions in one intervention raises the question whether it was kneeling astride the arm or placing the hands between the nipples, or both, that resulted in less abdominal hand placements. This study design does not answer this question, as we decided to test a new instruction set, not just a single instruction.

Development of these new instructions was an iterative process. In a previous simulation study we observed that many participants knelt down by the lower part of torso, when given the instructions “kneel down by the chest”. The majority of these participants also compressed too low on the chest [17]. To prevent this, we used the patient’s arm as landmark to position the rescuer better. While sitting astride the arm, the distance to lower half of sternum and to patient airways is short and this allow rescuers to remain in the same position and more easily alternate between compressions and ventilations without moving sideways.

Neither inter-nipple line nor center of the chest are clearly visible landmarks with three layers of clothing. Test subjects had to use their perception of these landmarks to guide themselves. Yeong reported less variability using internipple line as landmark compared to center of the chest, when identifying the location on a photo of dressed patients [22]. In addition, we observed participants in our previous simulation study who understood the center of the chest to be the same as the center of the torso. We judged that nipple line is a more specific instruction. To reduce variability and avoid too low hand placement, we selected internipple line as landmark for hand placement.

### “Internipple line is unreliable”

ERC Guidelines 2010 [12] states that internipple line is unreliable based on studies from Shin [24] and Kusunoki [25], and therefore lower half of sternum should be used in guidance for hand placement. Shin reports that internipple line location varies based on CT scans of adults. Kusunoki compared hand size (width) with lengths of sternum, and found that in overall 50% of the cases, the heel of the hand would extend to the xiphoid process, but only 3 out of 506 female patients had an internipple line crossing the xiphoid process or abdomen. Their validation study also demonstrated that the combination of female rescuer and male patient, which is the most likely combination in out of hospital cardiac arrests, [26,27] gave no cases of hand deviation into epigastrium [25]. Using an average female hand palm size of 9.8 ± 0.8 cm, as reported by Kusunoki [25], 10/18 participants from our reference group, placed their hands close to the xiphoid process and would overlap with the epigastrium, compared to only 3/18 of the intervention group. Both Kusunoki and Shin reports that the internipple line crosses the lower half of sternum. This justifies that “between the nipples” can be used in instructions.

### Time to first chest compression and between compression series

The time from scenario start until hand placement was not recorded, and it is reasonable to assume that the delay before the first compression might be longer with the intervention instructions than in the control group. Larsen and co-workers estimated a drop in survival by 2.3% per one minute delay to begin CPR [28]. But quality of CPR seems to matter more: In the studies by Gallagher [29], Van Hoeyweghen [30] and Wik [31], patients who received competent CPR were more than three times more likely to survive compared to those who received not-competent CPR. Based on this, it is reasonable to spend some extra seconds in the beginning to help ensure better quality CPR by avoiding abdominal compressions.

We did not test the effectiveness of kneeling astride the arm if a single rescuer performs both chest compressions and ventilations. This should reduce the need for repositioning between compression and ventilation attempts and thereby decrease time between compression series, but this needs to be further investigated.

### Limitations

The study was performed with only one adult playing the role of the patient, and in a larger study it would be beneficial to include several adults of different size and both genders.

The optimal hand placement for chest compressions is still unknown. A pilot study by Qvigstad evaluating ETCO_2_ as a surrogate marker for cardiac output during CPR on patients indicates that there is no specific hand placement that gives optimal cardiac output for all patients [32]. The purpose of our study was to test if a new set of instructions would avoid lay people placing their hands in epigastrium.

The translation of the instructions from one language to another might give semantic differences. To ensure the instructions are effective, they need to be validated, recognizing that culture and language influence our interpretation of instructions.

We tested instructions for initial hand placement only. It is unknown whether the participants would have maintained their hand placement during chest compressions or if a more cephalad hand placement would cause more shallow compressions. This should be further investigated.

## Conclusion

New pre-arrival instructions where the patient’s arm and nipple line were used as landmarks resulted in less caudal hand placements and none in the abdominal region.

## Competing interests

Birkenes receive research scholarships provided by the Norwegian Research Council. Birkenes and Myklebust are employees at Laerdal Medical. Kramer-Johansen receives financial research support from Laerdal Medical. The study was sponsored by Laerdal Medical, Stavanger, Norway.

## Authors' contributions

All authors participated in the study design. TSB and HM collected the data; TSB performed the statistical analysis and drafted the manuscript. All authors have critical reviewed the manuscript, and the study was supervised by JKJ. All authors read and approved the final manuscript.
